# CaMK II Inhibition Attenuates ROS Dependent Necroptosis in Acinar Cells and Protects against Acute Pancreatitis in Mice

**DOI:** 10.1155/2021/4187398

**Published:** 2021-11-17

**Authors:** Qingtian Zhu, Lu Hao, Qinhao Shen, Jiajia Pan, Weili Liu, Weijuan Gong, Lianghao Hu, Weiming Xiao, Mei Wang, Xinnong Liu, Yanbing Ding, Guotao Lu

**Affiliations:** ^1^Pancreatic Center, Department of Gastroenterology, The Affiliated Hospital of Yangzhou University, Yangzhou University, Yangzhou, China; ^2^Department of Gastroenterology, Changhai Hospital, The Second Military Medical University, Shanghai, China; ^3^Institute of Gastroenterology, Affiliated Hospital of Yangzhou University, Yangzhou University, Jiangsu, China; ^4^Department of Gastroenterology, The First Affiliated Hospital, Zhejiang University, China; ^5^Department of Intensive Care Unit, The Affiliated Hospital of Yangzhou University, Yangzhou University, Yangzhou, China

## Abstract

As a calcium-regulated protein, CaMK II is closely related to cell death, and it participates in the development of pathological processes such as reperfusion injury, myocardial infarction, and oligodendrocyte death. The function of CaMK II activation in acute pancreatitis (AP) remains unclear. In our study, we confirmed that the expression of p-CaMK II was increased significantly and consistently in injured pancreatic tissues after caerulein-induced AP. Then, we found that KN93, an inhibitor of CaMK II, could mitigate the histopathological manifestations in pancreatic tissues, reduce serum levels of enzymology, and decrease oxidative stress products. Accordingly, we elucidated the effect of KN93 in vitro and found that KN93 had a protective effect on the pancreatic acinar cell necroptosis pathway by inhibiting the production of ROS and decreasing the expression of RIP3 and p-MLKL. In addition, we identified the protective effect of KN93 on AP through another mouse model induced by pancreatic duct ligation (PDL). Together, these data demonstrated that CaMK II participates in the development of AP and that inhibiting CaMK II activation could protect against AP by reducing acinar cell necroptosis, which may provide a new idea target for the prevention and treatment of AP in the clinic.

## 1. Introduction

Acute pancreatitis (AP) is one of the most common and potentially devastating complications of digestive diseases, with a high mortality, great variability in severity, and high health-care costs [1–3]. Most clinical patients have mild and reversible disease, which is called mild acute pancreatitis (MAP). However, some of them may progress to severe acute pancreatitis (SAP), accompanied by extensive necrosis of pancreatic acinar cells, the activation and release of enzymes, and infiltration by a large number of inflammatory mediators [4, 5], which could induce a systemic inflammatory response and multiple organ failure, with a high mortality [6]. Animal experiments showed that inhibition of acinar cell necrosis could reduce the inflammatory response during AP [7, 8]. In the early stage of AP, effective reduction of acinar cell necrosis is the key to reducing the degree of inflammation and improving the prognosis of AP.

Increasing numbers of researchers have shown that necroptosis, a new concept of necrosis, is regulated by the RIP kinase family, including receptor interacting protein 3 (RIP3) and downstream molecule lineage kinase domain-like protein (MLKL), which are involved in the regulation of several inflammatory processes, including myocardial infarction [9], ischemia-reperfusion injury [10], inflammatory bowel disease [11], and chronic obstructive pulmonary disease [12]. Acinar cell necroptosis plays an important role in AP [13–15] and is related to the development of AP [16]. Notably, RIP3 deficiency or p-MLKL deletion could significantly reduce the acinar cell necroptosis induced by caerulein in AP mice [17, 18].

Ca^2+^/calmodulin-dependent protein kinase II (CaMK II), as a fundamental molecule in calcium signaling, is a heteromeric serine/threonine-specific protein kinase [19, 20]. The activated CaMK II works as a sensor of reactive oxygen (ROS) increments and correlated with a sustained kinase activity, then differentially localizing to regulate specific events [21, 22]. It has been shown that CaMK II is widely distributed in mammals, and it is highly expressed in the myocardium, brain, and kidney, where it regulates calcium homeostasis, enhances learning and memory, and maintains membrane stability, among other physiological functions [23]. However, continuous activation of CaMK II could aggravate myocardial necroptosis, subsequent malignant cardiac remodeling, and ultimately, sudden myocardial infarction via the necroptosis pathway. Inhibiting the expression and activation of CaMK II could improve the above pathological processes and reduce cell necroptosis [9, 24].

The function of CaMK II activation in AP is unclear. KN93 is a CaMK-binding specific antagonist that can reversibly and competitively inhibit CaMK II activity. KN93 has roles in Parkinson's disease, cerebral ischemia, hypoxia, and cancer pain [25, 26]. In the current article, we first confirmed that CaMK II was significantly increased in pancreatic acinar cells during AP, which is consistent with the expression of RIP3. We further verified that KN93 had dramatic effectiveness in two AP mouse models and that it decreases the expression of necrotic complexes (RIP3 and p-MLKL) in two cell lines, providing a new target for the prevention and treatment of AP in the clinic.

## 2. Materials and Methods

### 2.1. Chemicals and Reagents

KN93 was obtained from Selleck (Selleckchem, Texas, USA); caerulein, cholecystokinin (CCK), and DHE were purchased from Sigma (Sigma-Aldrich, St. Louis, MO, USA); anti-CaMK II antibody, anti-p-CaMK II antibody, anti-RIP3 antibody, anti-pMLKL antibody, anti-CD68 antibody, and anti-myeloperoxidase (MPO) were purchased from Abcam (Cambridge, UK); anti-GAPDH antibody was purchased from Sigma; and anti-RIP3 antibody was obtained from Santa Cruz Biotechnology (Santa Cruz, CA, USA). The anti-rabbit and anti-mouse secondary antibodies were purchased from Cell Signaling Technologies (Beverly, MA, USA). The LDH Cytotoxicity Assay Kit was purchased from Beyotime Biotechnology (Beijing, China); amylase kits were purchased from BioSino (BioSino BioTechnology & Science Inc., Beijing, China), and lipase kits were purchased from Nanjing Jiancheng (Nanjing Jiancheng Corp, Nanjing, China). Hoechst 33342 and propidium iodide (PI) were purchased from KeyGEN BioTECH, Nanjing, China. All flow cytometry-related antibodies were purchased from BioLegend.

### 2.2. Cell Culture and Stimulation

For the in vitro experiments, the mouse pancreatic acinar 266-6 cell line was obtained from ATCC (Manassas, VA, USA) and incubated in DMEM containing 10% fetal bovine serum (Gibco, Thermo Fisher Scientific), 100 U/ml penicillin, and 100 mg/ml streptomycin at 37°C with 5% CO_2_ in regular air. The 266-6 cells were incubated with different doses of KN93 (0.5, 1, and 2 *μ*M) for 30 min and then treated with 5 *μ*M CCK for 12 h as a cell injury model of AP. Finally, the cells were collected for subsequent experiments. In the control group, the 266-6 cells were only incubated with 0.1% DMSO.

Primary pancreatic acinar cells were freshly and quickly isolated from C57BL/6 mice using collagenase IV digestion [27]. The cells were suspended in HEPES buffer and pretreated with KN93 (2 *μ*M) for 30 min, followed by coincubation with CCK (1 *μ*M) for 2 h. Then, the cells were stained with Hoechst 33342 (50 *μ*g/ml) and propidium iodide (PI; 1 *μ*M). Necrotic cells were calculated as PI-positive cells divided by Hoechst 33342-stained cells and multiplied by 100%.

### 2.3. Animals

All studies were approved by the Science and Technology Commission of the Affiliated Hospital of Yangzhou University municipality, and all methods were carried out in accordance with the Principles of Laboratory Animal Care (NIH publication No. 85Y50, revised 1996). C57BL/6 male mice weighing 20-25 g were purchased from GemPharmatech Co., Ltd., Nanjing, China, and housed in a temperature-controlled, appropriate humidity animal facility with 12 h light-dark cycles. All mice had unlimited access to water and commercial food.

### 2.4. Construction of the AP Model and KN93 Administration In Vivo

To establish the AP model, male mice were induced by intraperitoneal (i.p.) injection of caerulein (100 *μ*g/kg, at intervals of 1 h, 7 times in total). Another AP model was induced by pancreatic duct ligation (PDL). After intraperitoneal injection of sodium pentobarbital (50 mg/kg), 1-2 cm longitudinal incisions were made in the abdomen of the mice to expose the abdominal cavity, and the duodenum was turned over to expose its distal side. At 1 cm above the duodenal papilla, blunt dissection of the tissue around the pancreatic duct was performed, and the tissue was ligated with a silk thread. Complete obstruction of the pancreatic duct was used to simulate biliary pancreatitis caused by cholelithiasis. After ligation, the abdominal cavity of the mice was layered and closed. All of the mice were placed on a 37°C constant temperature heating table for 90 min to recover from the surgery [28].

### 2.5. KN93 Administration In Vivo and Sample Collection

KN93 was dissolved in 100% DMSO to prepare a stock solution and then diluted in 5% DMSO solution/95% PBS before use. To verify the protective effect of KN93 against AP, KN93 (5, 10, and 20 mg/kg) was administered intraperitoneally 0 h before the administration of caerulein. All animals were anesthetized by intraperitoneal administration of sodium pentobarbital (50 mg/kg) before sacrifice. Blood samples were collected for ELISA measurement and amylase and lipase detection. The analysis was performed according to the kit instructions. Pancreatic and pulmonary tissues were harvested, and some were fixed immediately for histological analysis, and the rest were stored at -80°C for further investigation.

### 2.6. Histological Analysis

Pancreatic tissue samples were fixed in 4% phosphate buffered formaldehyde, embedded in paraffin blocks, stained with hematoxylin and eosin, and then examined with a light microscope. The histopathological scoring analysis of the pancreatic and pulmonary tissues was performed blindly according to our previously described methods [29].

### 2.7. Immunohistochemical Analysis

Mice were anesthetized and perfused transcranially with saline and 4% paraformaldehyde. The pancreas of the mice was obtained, fixed, embedded, and cut into 5 *μ*m thick sections. For IHC of RIP3 and p-MLKL, the slides were incubated overnight at 4°C in a humid chamber with antibodies against RIP3 (1 : 200 dilution) and p-MLKL (1 : 200 dilution) and then incubated with a biotinylated secondary antibody (1 : 500 dilution) for 60 min. Images were acquired through a light microscope.

### 2.8. Western Blot Analysis

For immunoblot analysis, the proteins from the pancreatic tissues were determined using a BCA protein kit (Thermo Fisher Scientific, MA, USA). Protein samples were subjected to 10% SDS-PAGE and then transferred to PVDF membranes. The membranes were blocked with 5% skim milk at room temperature for 2 h and then incubated overnight at 4°C with the following primary antibodies: anti-CaMK II (1 : 1000 dilution), anti-p-CaMK II (1 : 1000 dilution), RIP3 (1 : 1000 dilution), p-MLKL (1 : 1000 dilution), and GAPDH (1 : 2500 dilution) in blocking buffer. The membranes were washed with TBST (3 × 10 min) the next day and then incubated with the appropriate horseradish peroxidase-conjugated secondary antibodies for 1 h at room temperature. After washing, the protein bands were detected using the ECL Plus chemiluminescent system. Image intensity was analyzed with the ImageJ software.

### 2.9. Evaluation of Serum Enzymology and Enzyme-Linked Immunosorbent Assay (ELISA) Determination

The in vitro proliferation and quantitative detection of toxicity were determined by an LDH release kit. Briefly, exponentially growing 266-6 cells were seeded into 96-well microplates at a density of 5 × 10^3^ cells/well, followed by 12 h incubation and then assayed according to the instructions of the LDH Kit. Blood samples were collected at different time points for serum enzymology (amylase and lipase) and proinflammatory cytokine detection. The analysis was performed according to the kit instructions.

### 2.10. Measurement of ROS Generation in Pancreatic Tissues

The assays were performed as in our previously described methods [28]. Fresh tissues from the pancreas were embedded in optimal cutting temperature (OCT) compound, and then, the samples were cut into 7 mm sections. The tissues were incubated in the dark with DHE solution for 30 min at 37°C. Slides were placed in PBS (pH = 7.4) and washed 3 times, each time for 5 min. Then, the tissues were incubated with 4′,6-diamidino-2-phenylindole, dihydrochloride (DAPI, Wuhan Servicebio Technology Co., Ltd., Wuhan, China) solution at room temperature for 10 min and washed again. Finally, the slides were observed under a fluorescence microscope.

### 2.11. Oxidative Stress Product Detection

Briefly, pancreatic tissues were homogenized in PBS and then centrifuged (12,000 rpm, 4°C, 30 min) to obtain supernatant. CAT, MDA, and GSH levels were determined according to the manufacturer's instructions [30].

### 2.12. Pancreatic Leukocyte Isolation and Preparation

Pancreatic leukocytes were isolated using a collagenase digestion method for flow cytometry analysis [31]. In brief, the pancreas was cut into pieces and digested in FACS buffer containing 2 mg/ml collagenase type IV (Sigma-Aldrich). The tissues were incubated in a shaker at 37°C for 17-20 min and then vortexed at low speed for 20 s before passage through a 70 *μ*M filter. The above cells isolated from different tissues were subjected to RBC lysis if necessary prior to further staining or treatment.

### 2.13. Flow Cytometry

For surface staining, the murine cells were stained with Abs against the following: CD45.2 (clone 104), CD11b (clone M1/70), F4/80 (clone BM8), and Gr-1 (clone RB6-8C5). Cells were washed, stained with surface markers, acquired on a Beckman DxFlex and analyzed with CytExpert for DxFLEX.

### 2.14. Statistical Analysis

Statistical analysis was performed by the GraphPad Prism 6 software (GraphPad, San Diego, CA, USA). The *t*-test was used to analyze differences between two groups, and one-way ANOVA was used to evaluate the statistical significance among groups. The results are presented as the mean ± SEM (standard error of the mean), and *P* < 0.05 was considered statistically significant (two-tailed). At least three independent experiments were carried out for the statistical comparisons.

## 3. Results

### 3.1. CaMK II Was Persistently Highly Expressed in the Pancreatic Tissues of AP Mice

**Figure 1:**
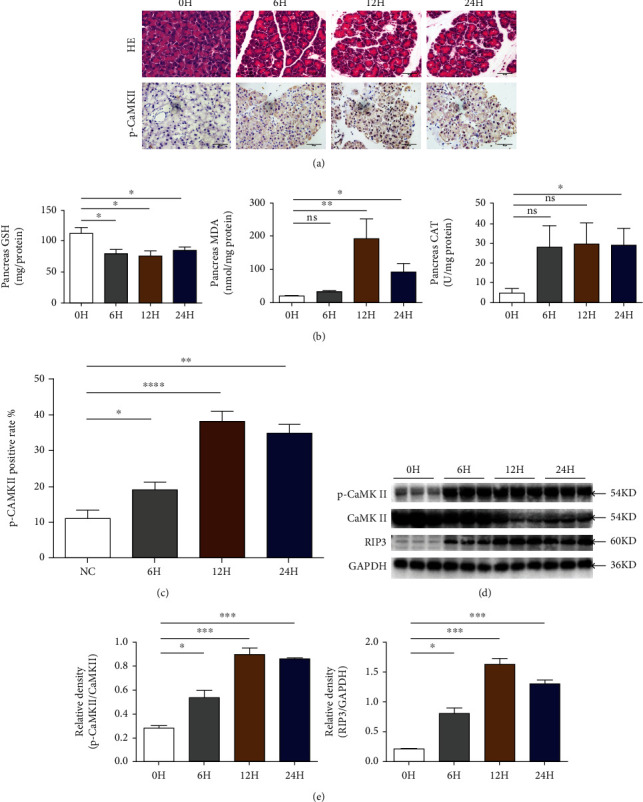
CaMK II was persistently highly expressed in the pancreatic tissues of AP mice. (a) Representative HE staining and immunohistochemistry images for p-CaMKII expression of pancreatic tissues in magnifications 400x. Scar bar = 50 *μ*M. *N* = 6 each time point. (b) Levels of oxidative stress products (CAT, MDA, and GSH) of pancreas. *N* = 6 each group. (c) p-CaMK II positive rate of immunohistochemistry images in the pancreas. (d) Protein levels of p-CaMK II and RIP3 in pancreatic tissues were analyzed by Western blotting. (e) CaMK II and GAPDH were used as a control for protein loading, respectively. (b–e) *N* = 3 each time point. ^∗^*P* < 0.05, ^∗∗^*P* < 0.01, ^∗∗∗^*P* < 0.001, and ^∗∗∗∗^*P* < 0.0001.

As shown in Figure 1(a) and SFigure 1, after caerulein administration, we observed that pancreatic injury began at 6 h and reached a peak at 12 h, which mainly represented edema, inflammatory cell infiltration, and acinar cell necrosis. Serum amylase and lipase, which are common biochemical markers of AP, increased gradually until 12 h and then declined at 24 h. In addition, as shown in Figure 1(b), there was an increase in the MDA and CAT levels and a decrease in the GAH level in pancreatic tissues of AP.

Immunohistochemistry (IHC) staining was used to detect that the expression of p-CaMK II in pancreatic tissues, and it was significantly elevated after AP onset, as shown in Figures 1(a) and 1(c). In addition, the expression of p-CaMK II protein by Western blot detection presented the same trend as IHC staining. Accordingly, the expression of CaMK II was decreased in pancreatic acinar cells after caerulein stimulation, as shown in Figures 1(d) and 1(e).

### 3.2. KN93 Mitigated the Severity of Caerulein-Induced AP in Mice

**Figure 2:**
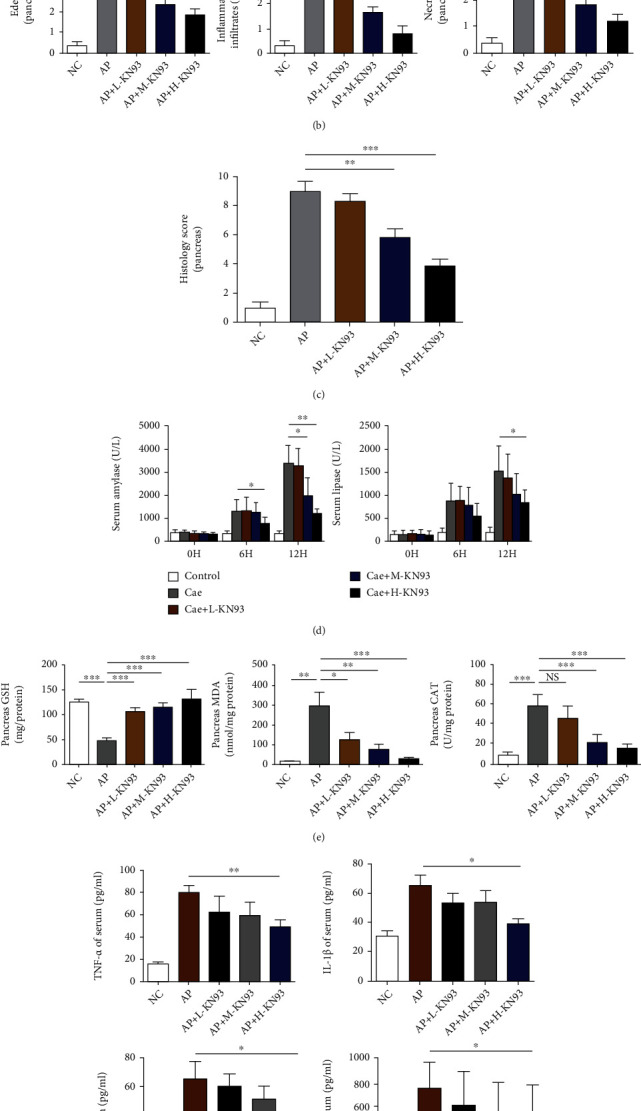
KN93 mitigated the severity of caerulein-induced AP in mice. (a) Representative HE staining of pancreatic tissues in magnifications 100x and 400x. Scar bar = 50 *μ*M. (b, c) The pathological scores of pancreatic tissues. (d) Serum levels of amylase and lipase. (e) The levels of GSH, MDA, and CAT in the pancreatic tissues. (f) The serum levels of TNF-*α*, IL-1*β*, IL-6, and MCP-1 was detected by ELISA. *N* = 7 each group. ^∗^*P* < 0.05 and ^∗∗^*P* < 0.01. L, M, and H represent low-dose (5 mg/kg), medium-dose (10 mg/kg), and high-dose KN93 (20 mg/kg).

Whether KN93 has a protective effect against AP has not previously been reported. Based on the high expression of p-CaMK II in caerulein-induced AP, the mice were sacrificed 12 h after the administration of caerulein. As shown in Figures 2(a)–2(d), the severity of AP was significantly reduced after KN-93 administration, accompanied by decreased inflammatory cell infiltration, decreased serum levels of amylase and lipase, and decreased acinar cell necrosis. All of the results indicated that the protective effect of KN93 on AP is dose-dependent, and the highest dose of KN-93 had the best effect without toxic side effects on the mice, as shown in SFigure 2. In addition, the levels of oxidative products (MDA, CAT, and GSH), as shown in Figure 2(e), were detected in the pancreatic tissues, and we found that MDA and CAT were decreased and GSH was significantly increased after KN93 treatment compared with AP groups. The dynamic changes of oxidative stress products may be accompanied by the levels of proinflammatory cytokines, such as IL-1*β*, IL-6, TNF-*α*, and MCP-1 on AP. Accordingly, the results showed that these serum inflammatory mediators were increased approximately 2-3-fold compared with those in the NC group, and they could be attenuated by high-dose KN93 (H-KN93) administration, as shown in Figure 2(f), which was in accordance with the degree of pancreatic injury. In view of this, H-KN93 (20 mg/kg) was adopted to carry out functional and mechanistic research in the following experiments.

### 3.3. KN93 Decreased Macrophages and Neutrophils in Pancreatic Tissues of the AP Mouse Model

**Figure 3:**
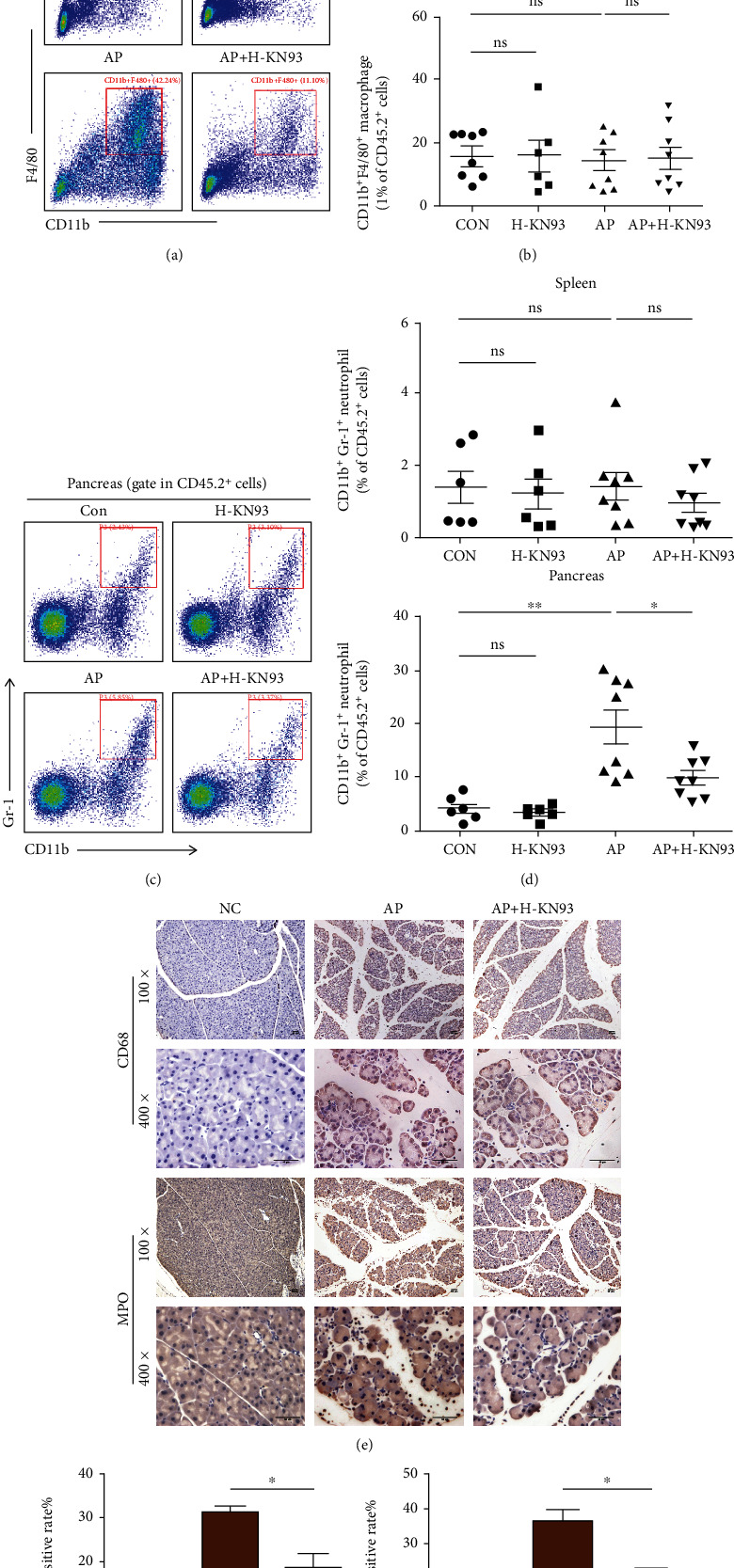
KN93 decreased macrophages and neutrophils in pancreatic tissues of the AP mouse model. (a–d) Leukocytes from pancreas and spleen were isolated for staining and flow cytometry analysis. Representative flow cytometry gating of pancreatic macrophages (a) and neutrophils (c) is shown. (b, d) The percentages of CD11b ^+^F4/80^+^ macrophages and CD11b^+^Gr-1^+^ neutrophils from pancreas and spleen are shown (mean ± SEM) as scatter plots. *N*≧6 each group. (e) Representative immunohistochemistry images for myeloperoxidase (MPO) and CD68 in the pancreas. Scar bar = 50 *μ*M. (f) The frequencies of MPO positive rate and CD68 positive rate in pancreas. *N* = 3 each group. ^∗^*P* < 0.05 and ^∗∗^*P* < 0.01. H represents high-dose KN93 (20 mg/kg).

Increasing amounts of evidence support the importance of infiltrating leukocytes, especially macrophages and neutrophils, in the development of AP pathogenesis [31]. We established an AP model induced by caerulein and isolated leukocytes from the pancreas and spleen. As shown in Figures 3(a)–3(d), we observed that the number of activated macrophages (CD11b^+^F4/80^+^) and neutrophils (CD11b + GR-1^+^) in the pancreas was significantly increased in AP mice compared with the control group, while the number of leukocytes in the spleen was not different among the four groups. Unsurprisingly, the number of infiltrating leukocytes could be reduced by KN93 administration to AP mice, while KN93 injection had no effect on the numbers or differentiation of the leukocytes in the mice.

MPO and CD68 are widely used as biomarkers of activated neutrophils and macrophages. IHC examination of MPO and CD68 in pancreatic tissues was used to further assess pancreatic tissue inflammatory cell infiltration. These results indicated that KN93 significantly decreased the infiltration of neutrophils and macrophages compared with that in the AP group (Figures 3(e) and 3 (f)), which was consistent with the flow cytometry results.

### 3.4. KN93 Reduced Acinar Cell Necroptosis and Decreased the Production of ROS in Acinar Cells in AP Mice

**Figure 4:**
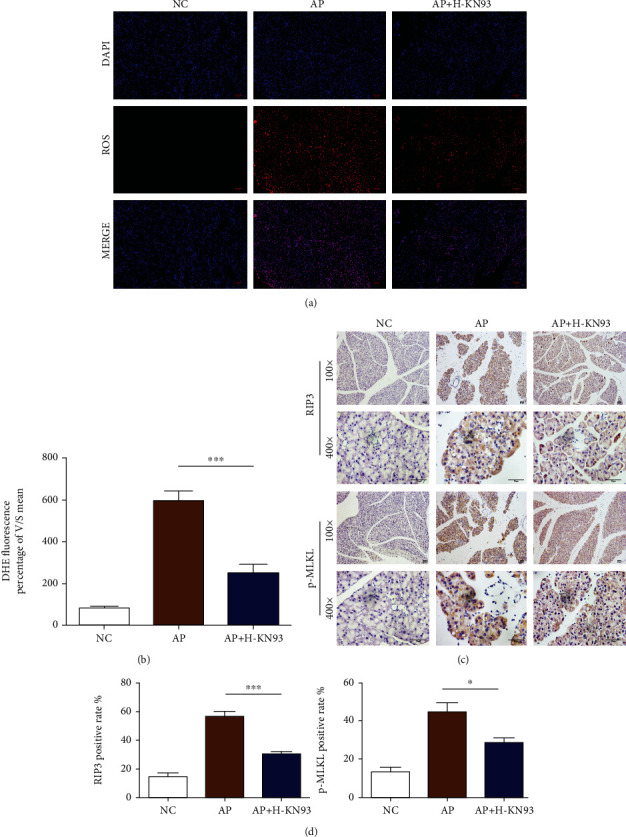
KN93 reduced acinar cell necroptosis and decreased the production of ROS in acinar cells in the AP mice. (a) Representative immunohistochemistry images for RIP3 and p-MLKL expression in the pancreas. Scar bar = 50 *μ*M. (b) Representative immunofluorescence image of DHE in magnification 200x. Scar bar = 50 *μ*M. (c) Densitometric analysis of DHE fluorescence. (d) RIP3 and p-MLKL positive rate of pancreatic acinar cells. *N* = 3 each group. ^∗^*P* < 0.05, ^∗∗^*P* < 0.01, and ^∗∗∗^*P* < 0.001. H represents high-dose KN93 (20 mg/kg).

The production of ROS and the expression of RIP3 and p-MLKL have been considered to be associated with necroptosis. To further investigate the potential functions of KN93 in AP, we detected the production of ROS and the expression of RIP3 and p-MLKL in pancreatic tissues. DHE staining showed that the production of ROS was remarkably increased during AP, while H-KN93 reversed this increase (Figures 4(a) and 4(b)). As shown in Figures 4(c) and 4(d), IHC staining illustrated a significant increase in the expression of RIP3 and p-MLKL in pancreatic acinar cells, which was reduced after H-KN93 treatment. All of these data indicated that KN93 could inhibit the necroptosis pathway in acinar cells.

#### 3.4.1. The Protective Effect of KN93 on Pancreatic Acinar Cells In Vitro

**Figure 5:**
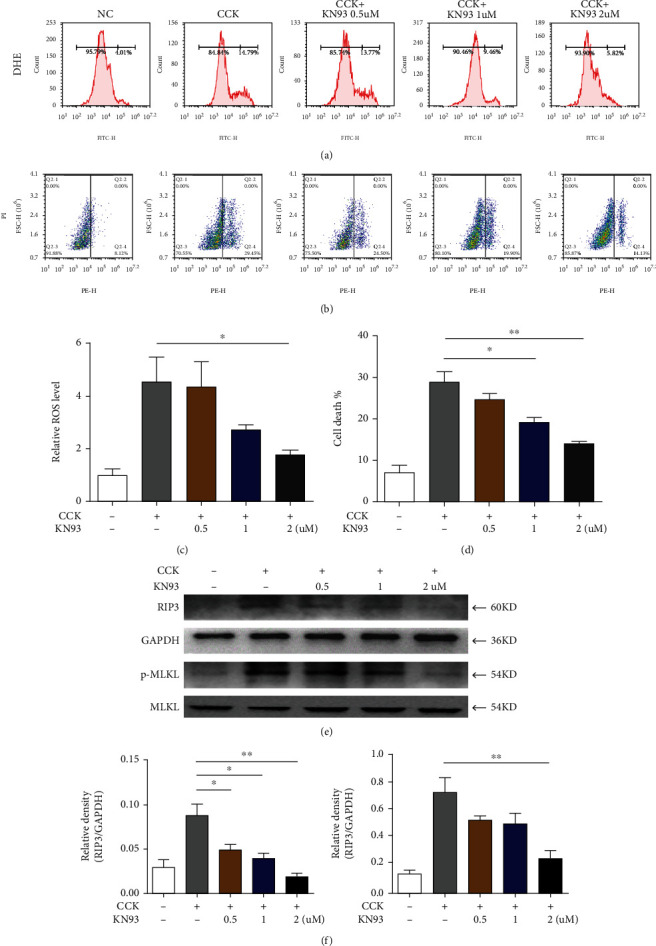
Effect of KN93 on 266-6 cells in vitro. (a) PI was detected by flow cytometry. (b) The reactive oxygen species (ROS) in pancreatic tissues were detected by DHE fluorescent probe. (c) The relative cell death level of 266-6 cells. (d) The relative ROS level of 266-6 cells. (e) Protein levels of RIP3 and p-MLKL in 266-6 cells were analyzed by Western blotting. (f) Relative protein expression of RIP3 and p-MLKL; GAPDH and MLKL were used as control for protein loading, respectively. ^∗^*P* < 0.05 and ^∗∗^*P* < 0.01. CCK: cholecystokinin octapeptide.

To further clarify the protective effect of KN93, the 266-6 cell line was used to establish a cellular injury model by CCK. As shown in Figures 5(a)–5(c), KN93 significantly reduced ROS production and protected against cell necrosis. In addition, CCK caused acinar cell injury, with the main manifestations following a pronounced increase in RIP3 and p-MLKL protein expression, which was decreased by KN93 (Figures 5(e)–5(f)).

**Figure 6:**
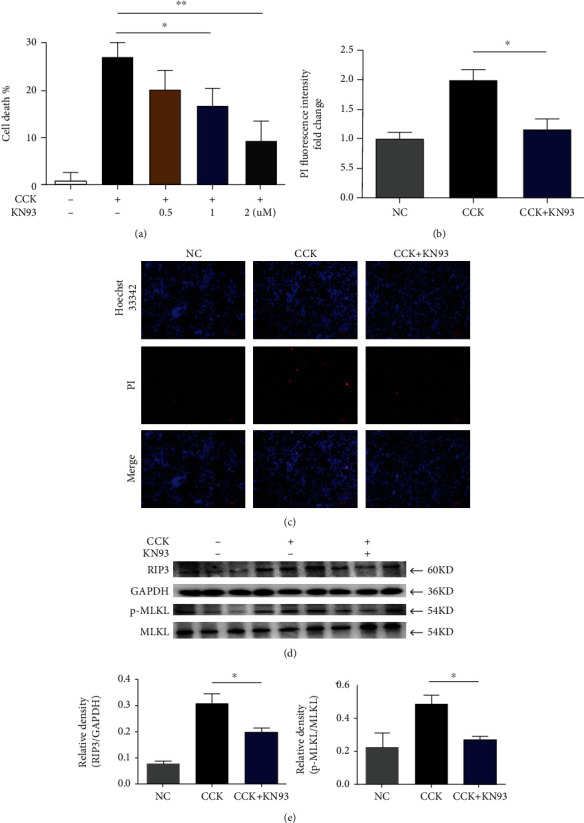
The protective effect of KN93 on pancreatic acinar cells in vitro. Freshly isolated mouse pancreatic acinar cells received prior incubation with KN93 only for 30 min. Then, cells were incubated with cholecystokinin (CCK; 1 *μ*M) for 1 h. (a) The relative cell death level of pancreatic acinar cells. (b) Cells were further stained with Hoechst 33342 and propidium iodide (PI) for determination of cell death by epifluorescence microscopy (100x). Scar bar = 50 *μ*M. (c) Densitometric analysis of PI fluorescence. (d) Protein levels of RIP3 and p-MLKL in pancreatic acinar cells were analyzed by Western blotting. (e) Relative protein expression GAPDH and MLKL were used as control for protein loading, respectively. ^∗^*P* < 0.05 and ^∗∗^*P* < 0.01. CCK: cholecystokinin octapeptide.

Furthermore, we validated the effectiveness of KN93 on isolated pancreatic acinar cells of mice and found that KN93 had a protective effect against acinar cell necrosis in different validation methods, as shown in Figures 6(a)–6(e).

#### 3.4.2. KN93 Mitigated the Severity of PDL-Induced AP and Prolonged Survival of Mice

**Figure 7:**
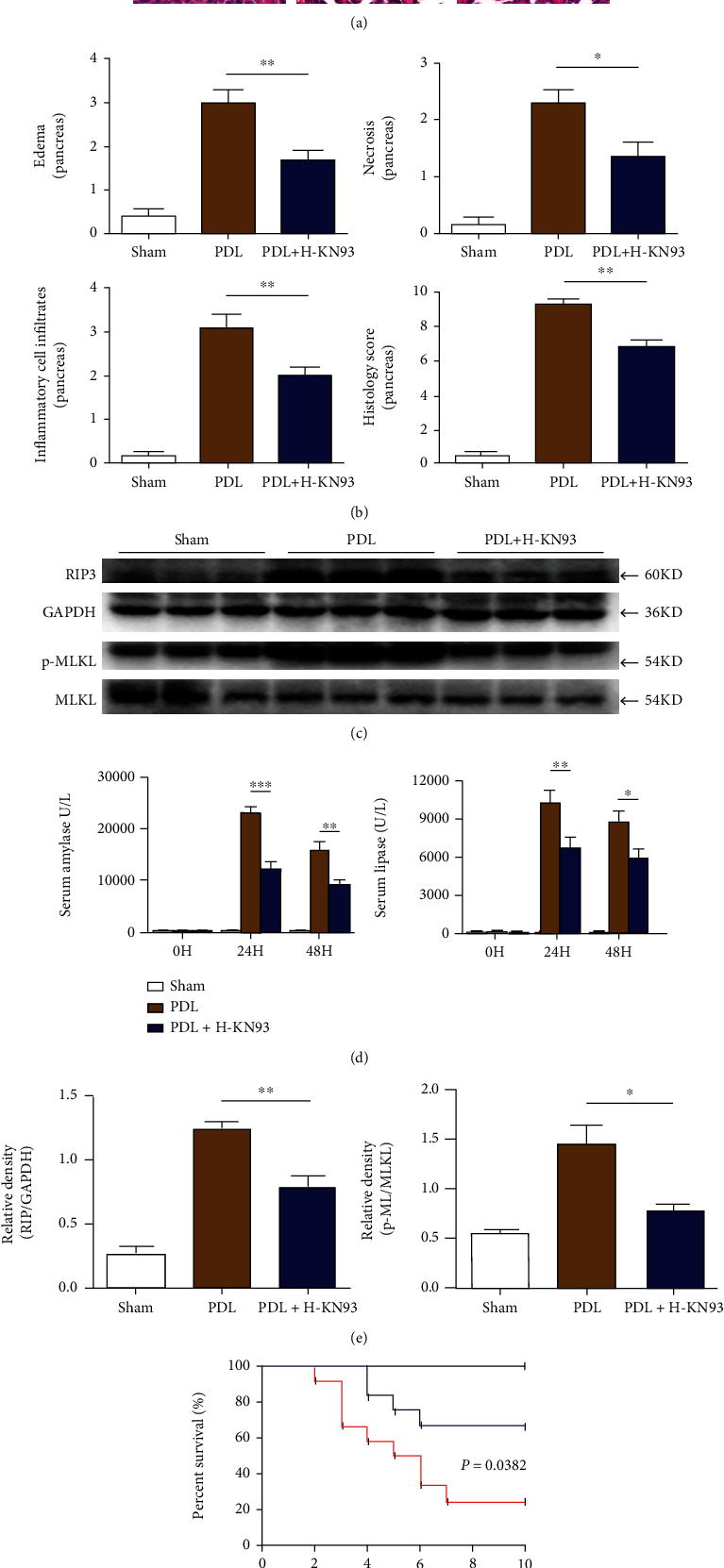
KN93 mitigated the severity of PDL-induced AP and prolonged survival of mice. (a) Representative HE staining of pancreas in SAP model induced by PDL in magnifications 100x and 400x. Scar bar = 50 *μ*M. (b) Histological scores of pancreas in SAP model induced by PDL. (c) Protein levels of RIP3 and p-MLKL in pancreas were analyzed by Western blotting. (d) Serum levels of amylase and lipase in SAP model induced by PDL. (e) Relative protein expression GAPDH and MLKL were used as control for protein loading, respectively. (f) For survival studies, KN93 was applied to PDL mice 1 day to 4 days after surgery and survivorship curve of mice in SAP model induced by PDL. *N* = 12 each group. ^∗^*P* < 0.05, ^∗∗^*P* < 0.01, and ^∗∗∗^*P* < 0.001. PDL: pancreatic duct ligation. H represents high-dose KN93 (20 mg/kg).

We generated an additional AP mouse model (induced by pancreatic duct ligation as an SAP model) to observe the expression of RIP3 and p-MLKL (Figures 7(d) and 7(e)) and further confirm the protective effect of KN93 on AP. Unsurprisingly, all of the results were consistent, including the serum enzyme changes, the pancreatic injury, and the related acute lung injury (commonly used to assess SAP), and suggested that KN93 had a good protective effect against AP (Figures 7(a), 7(b), and 7(d), SFig 3). Most notably, KN93 treatment was applied to PDL mice at 1 day to 4 days (total 20 mg/kg) after modeling. As shown in Figure 7(f), Kaplan-Meier curves revealed a significant difference in the survival rate (*P* = 0.0382) between PDL + KN93 and PDL mice, which suggested that KN93 could reduce the long-term mortality of mice in the PDL-induced SAP model.

## 4. Discussion

In summary, our study confirmed that CaMK II was involved in the progression of AP and validated that its inhibitor KN93 has a protective influence both in vivo and in vitro. It is possible that KN93 may be a promising drug for the treatment and prevention of AP in clinical practice in the future.

Acute pancreatitis (AP) is an acute noninfectious inflammatory disease involving abnormal inflammation of the pancreas. The pathogenesis of AP remains unclear. Current lines of evidence indicate that the pathogenesis and prognosis of AP might be associated with the oxidative stress-related acinar cell necrosis, and antioxidation could reduce pancreatic necrosis. Our previous studies found that oxidative stress products showed dynamic changes in pancreatic tissues of mice with AP. Additionally, the caerulein-induced AP model and PDL-induced SAP are essentially caused by hypersecretion of digestive enzymes of necrosis acinar cells [32, 33]. Inhibition of this could effectively reduce the inflammatory response and relieve the progression of AP [15]. Hence, it is vitally important to search for effective and therapeutic target drugs to reduce acinar cell necrosis both in basic research and in the clinic.

CaMK II is a family of serine/threonine kinases that are regulated by the Ca2^+^/calmodulin complex. CAM is a highly conserved calcium target protein, and it has no enzymatic activity under normal conditions. Once the cells are damaged by various stresses, the increased intracellular calcium content combined with CAM and subsequent activation of the downstream target protein CaMK II [19, 20]. Existing studies have confirmed that an increase of ROS production leading to CaMK II hyperactivity and perturbation in Ca2+ handing, and it has been reported to regulate and control inflammation-related diseases, such as reperfusion injury, myocardial infarction, and oligodendrocyte death [34–36]. Moreover, it is clear that KN93, as a reversible and competitive inhibitor of CaMK II, has been involved in reducing cell necroptosis in several contexts, such as osteoclast genesis and cerebral arteries [37, 38]. In the present study, the role of CaMK II in AP was confirmed. We demonstrated that the expression of CaMK II was significantly upregulated in the pancreas of AP mice and in injured acinar cells (both in the 266-6 cell line and in primary pancreatic acinar cells). Thereafter, the protective effect of KN93 on AP acinar cell necrosis was verified both in vivo and in vitro. All of these results indicated that CaMK II participated in pancreatic acinar cell necroptosis and that inhibition of the activation of CaMK II may be a new clinical target for the prevention and treatment of AP.

Recent studies have shown that the pathways regulating necroptosis are important in injured cells affected by inflammatory diseases, and the RIP3-MLKL-containing necrosis death complex (necrosome) is considered a key marker of the necrosis pathway [39, 40]. RIP3 or MLKL knockout mice have been shown to have reduced necroptosis of pancreatic tissue in a caerulein-induced AP model compared to wild-type mice [17, 18]. CaMK II is a RIP3/MLKL substrate and delineates the myocardial and intracerebral hemorrhage necroptosis pathways [9, 41]. However, the role of CaMK II activation and its involvement in cell death-related pathway in AP was still unclear. In this study, we found that the production of ROS and the expression of RIP3 and p-MLKL were significantly upregulated in the pancreas of mice with caerulein-induced AP, and these phenomena were blocked by KN93 intervention. We observed the same protective effect in vitro (two cell lines), which was consistent with the results of the above animal experiments. All data show that CaMKII plays a crucial role in the occurrence and progression of AP by mediating necroptosis in acinar cells.

It is well known that pancreatic necrosis is a risk factor for a poor prognosis of AP patients. The mortality of patients without pancreatic necrosis is only 2%, while that of patients with pancreatic necrosis is as high as 5-30% [9]. Accordingly, a decrease in mortality is a key indicator to explore the protective effects of drugs in basic AP studies. In the present study, a mouse model of SAP was established by PDL, accompanied by massive pancreatic necrosis and significant mortality. A clear phenomenon was observed in which the mortality of SAP mice decreased significantly after KN93 treatment, which is consistent with the result that KN93 protected against acute phase injury in AP. The mortality data further verified the protective effect of KN93 against AP.

## 5. Conclusion

**Figure 8:**
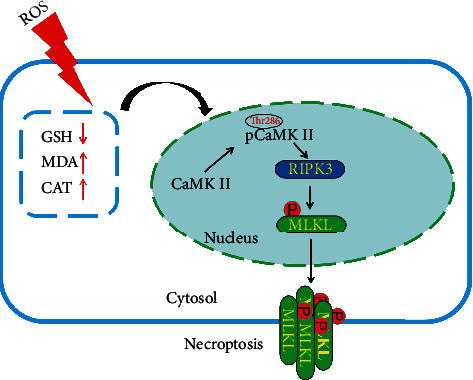
The mechanisms of CaMK II in damaged pancreatic tissues on AP mice.

In summary, our findings demonstrated that CaMK II plays an important role in AP and that inhibiting CaMK II may protect against AP by reducing the generation of ROS and modulating the necroptosis pathway (Figure 8).

## Data Availability

Our manuscript did not have publicly available data.

## Supplementary Materials

Supplementary Materials Supplementary Figure 1: dynamic changes of pathobiology and serum enzymology on AP mice induced by caerulein. (A) Histological scores of pancreas in AP model induced by caerulein. (B) Serum levels of amylase and lipase in AP model. Supplementary Figure 2: KN93 had no toxic side effects on mice. (A) Representative HE staining of pancreas, heart, lung liver, and kindey in magnifications 100x and 400x. Scar bar=50 *μ*M. (B) Serum levels of amylase and lipase. *N*=6 each group. H represent high-dose KN93 (20 mg/kg). Supplementary Figure 3: KN93 attenuated pancreatic-associated lung injury of mice with PDL-induced AP. (A) Representative HE staining of pancreas in SAP model induced by PDL in magnifications 100x and 400x. Scar bar=50 *μ*M. (B) Histological scores of lungs in SAP model induced by PDL. *N*=12 each group. ^∗^*P* < 0.05 and ^∗∗^*P* < 0.01. PDL: pancreatic duct ligation. H: high-dose KN93 (20 mg/kg).
