# Supplementary figures and images for: CaMK II Inhibition Attenuates ROS Dependent Necroptosis in Acinar Cells and Protects against Acute Pancreatitis in Mice
Source: Oxid Med Cell Longev. 2021 Nov 17;2021:4187398. doi: 10.1155/2021/4187398 (PMC8612788; doi:10.1155/2021/4187398)

**A**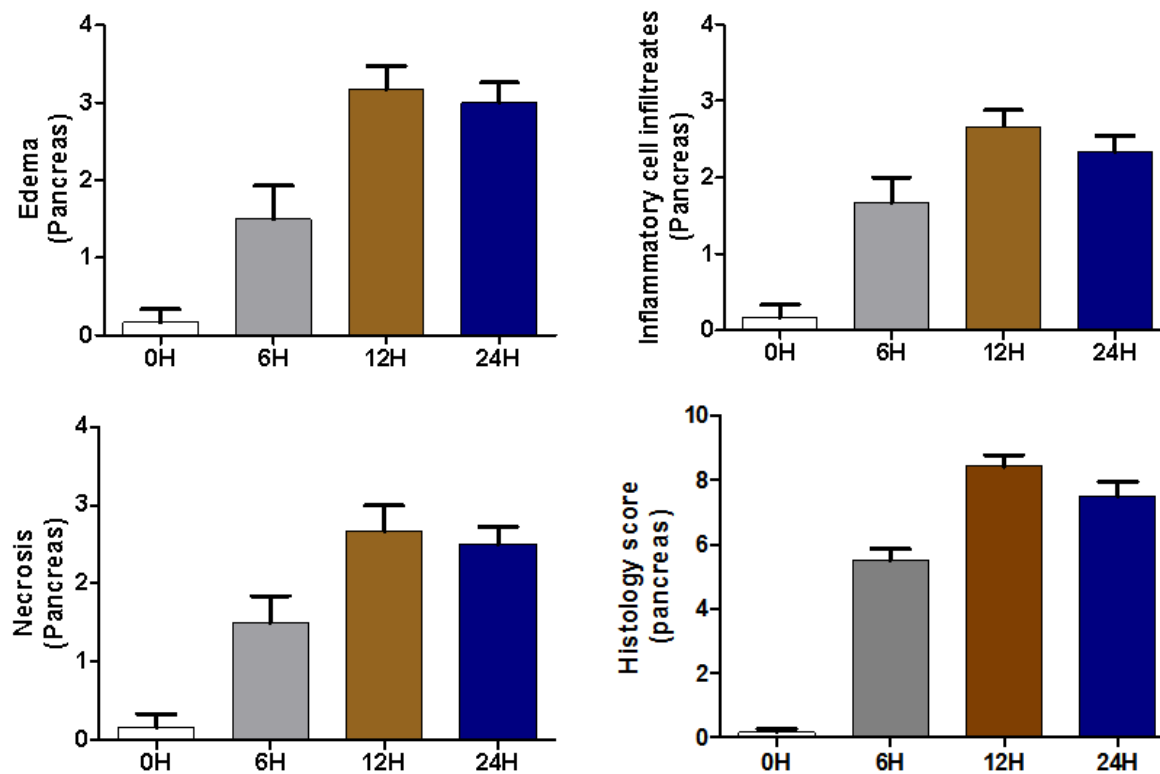**B**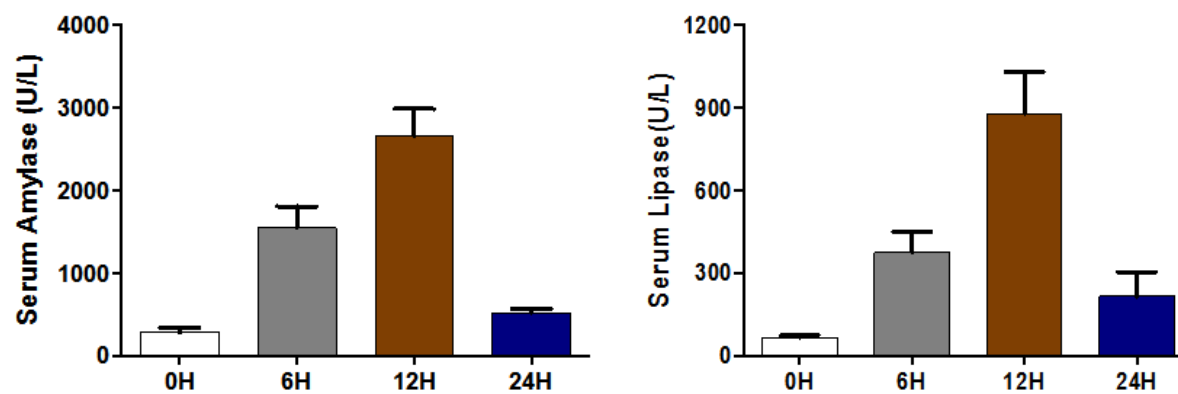

Supplement: Supplementary Materials — Supplementary Figure 1: dynamic changes of pathobiology and serum enzymology on AP mice induced by caerulein. (A) Histological scores of pancreas in AP model induced by caerulein. (B) Serum levels of amylase and lipase in AP model. Supplementary Figure 2: KN93 had no toxic side effects on mice. (A) Representative HE staining of pancreas, heart, lung liver, and kindey in magnifications 100x and 400x. Scar bar=50 μM. (B) Serum levels of amylase and lipase. N=6 each group. H represent high-dose KN93 (20 mg/kg). Supplementary Figure 3: KN93 attenuated pancreatic-associated lung injury of mice with PDL-induced AP. (A) Representative HE staining of pancreas in SAP model induced by PDL in magnifications 100x and 400x. Scar bar=50 μM. (B) Histological scores of lungs in SAP model induced by PDL. N=12 each group. ∗P < 0.05 and ∗∗P < 0.01. PDL: pancreatic duct ligation. H: high-dose KN93 (20 mg/kg). [file 4187398.f1.zip › sfgure1.pdf]

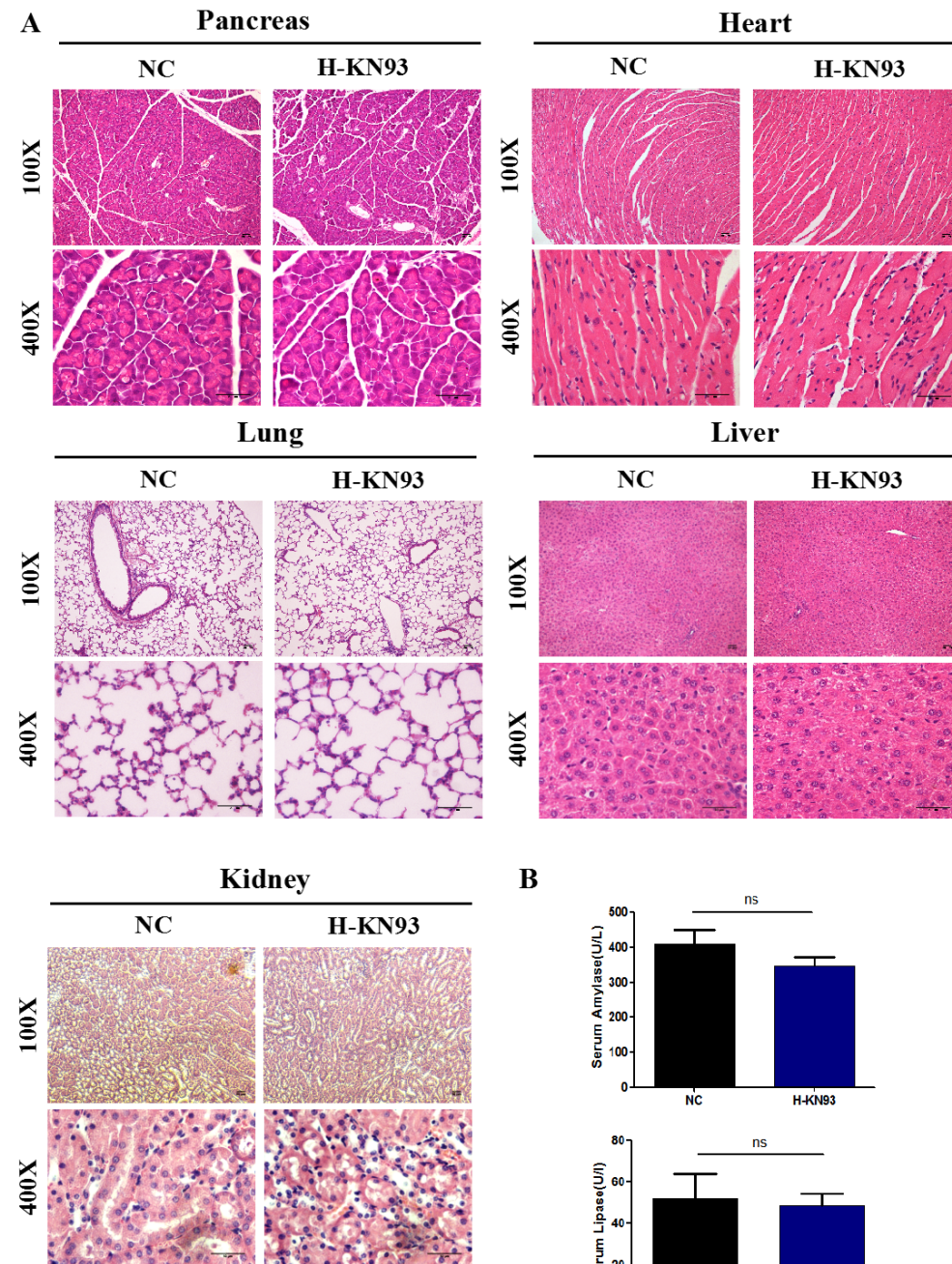

Supplement: Supplementary Materials — Supplementary Figure 1: dynamic changes of pathobiology and serum enzymology on AP mice induced by caerulein. (A) Histological scores of pancreas in AP model induced by caerulein. (B) Serum levels of amylase and lipase in AP model. Supplementary Figure 2: KN93 had no toxic side effects on mice. (A) Representative HE staining of pancreas, heart, lung liver, and kindey in magnifications 100x and 400x. Scar bar=50 μM. (B) Serum levels of amylase and lipase. N=6 each group. H represent high-dose KN93 (20 mg/kg). Supplementary Figure 3: KN93 attenuated pancreatic-associated lung injury of mice with PDL-induced AP. (A) Representative HE staining of pancreas in SAP model induced by PDL in magnifications 100x and 400x. Scar bar=50 μM. (B) Histological scores of lungs in SAP model induced by PDL. N=12 each group. ∗P < 0.05 and ∗∗P < 0.01. PDL: pancreatic duct ligation. H: high-dose KN93 (20 mg/kg). [file 4187398.f1.zip › sfigure2.pdf]

**A**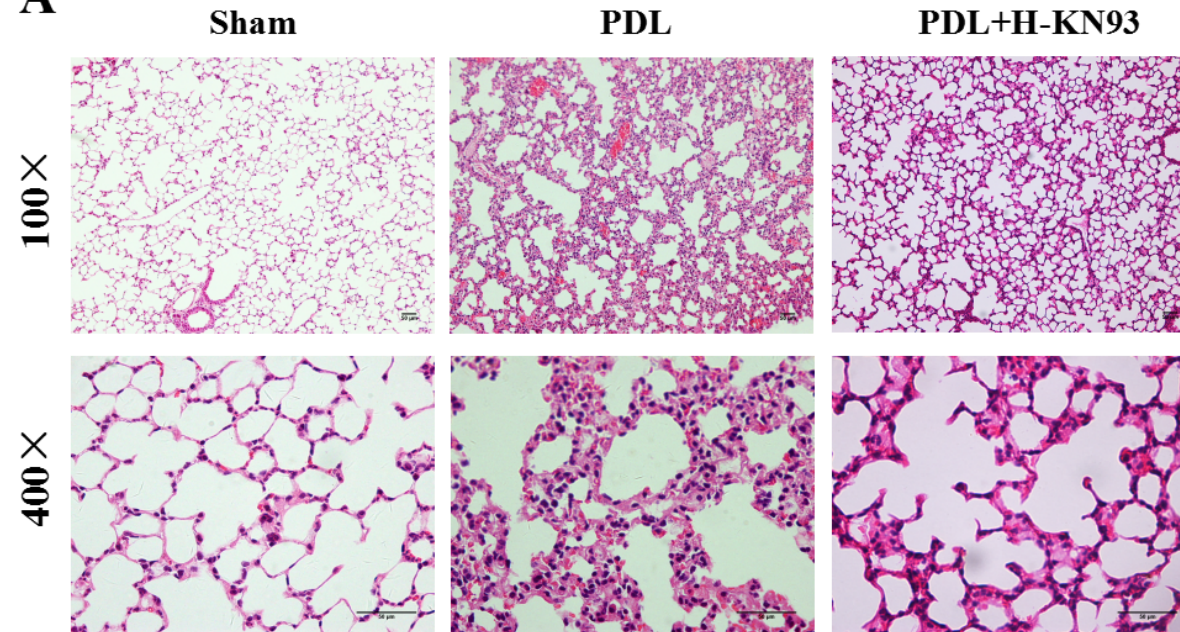**B**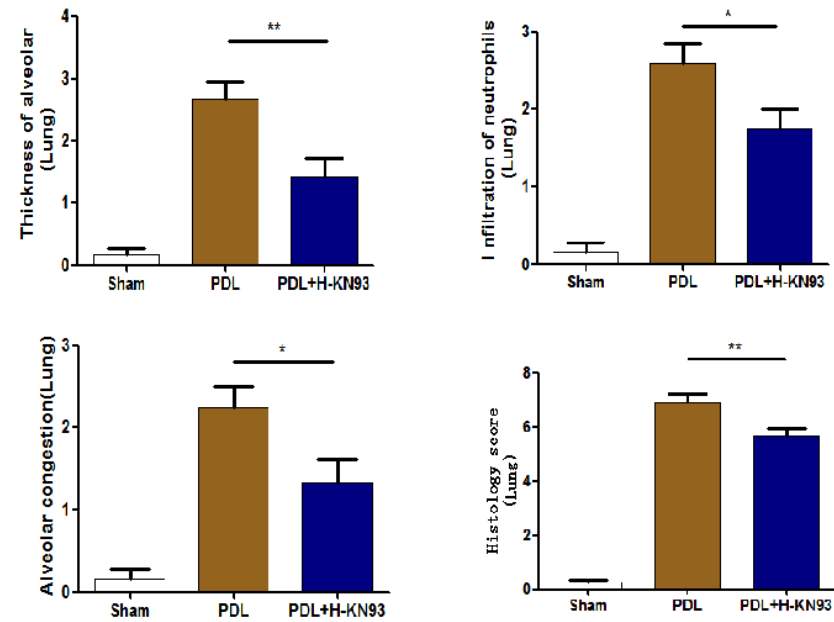

Supplement: Supplementary Materials — Supplementary Figure 1: dynamic changes of pathobiology and serum enzymology on AP mice induced by caerulein. (A) Histological scores of pancreas in AP model induced by caerulein. (B) Serum levels of amylase and lipase in AP model. Supplementary Figure 2: KN93 had no toxic side effects on mice. (A) Representative HE staining of pancreas, heart, lung liver, and kindey in magnifications 100x and 400x. Scar bar=50 μM. (B) Serum levels of amylase and lipase. N=6 each group. H represent high-dose KN93 (20 mg/kg). Supplementary Figure 3: KN93 attenuated pancreatic-associated lung injury of mice with PDL-induced AP. (A) Representative HE staining of pancreas in SAP model induced by PDL in magnifications 100x and 400x. Scar bar=50 μM. (B) Histological scores of lungs in SAP model induced by PDL. N=12 each group. ∗P < 0.05 and ∗∗P < 0.01. PDL: pancreatic duct ligation. H: high-dose KN93 (20 mg/kg). [file 4187398.f1.zip › sfigure3.pdf]
